# The Activation of AMPK/NRF2 Pathway in Lung Epithelial Cells Is Involved in the Protective Effects of Kinsenoside on Lipopolysaccharide-Induced Acute Lung Injury

**DOI:** 10.1155/2022/3589277

**Published:** 2022-03-18

**Authors:** Yue Yang, Zhen-tong Zhong, Yong-guang Xiao, Hong-bin Chen

**Affiliations:** ^1^Department of Orthopedics, Renmin Hospital of Wuhan University, Wuhan 430060, China; ^2^Department of Critical Care Medicine, Renmin Hospital of Wuhan University, Wuhan 430060, China; ^3^Department of Thoracic, Renmin Hospital of Wuhan University, Wuhan 430060, China; ^4^Department of Pulmonary and Critical Care Medicine, Renmin Hospital of Wuhan University, Wuhan 430060, China

## Abstract

The disorder of mitochondrial dynamic equilibrium of lung epithelial cell is one of the critical causes of acute lung injury (ALI). Kinsenoside (Kin) serves as an active small-molecule component derived from traditional medicinal herb displaying multiple pharmacological actions in cancers, hyperglycemia, and liver disease. The objective of this study was to investigate the effects of Kin on lipopolysaccharide- (LPS-) induced ALI and further explore possible molecular mechanisms. Kin was administered orally (100 mg/kg/day) for 7 consecutive days before LPS instillation (5 mg/kg). After 12 hours, pathological injury, inflammatory response, and oxidative stress were detected. The results demonstrated that Kin significantly alleviated lung pathological injury and decreased the infiltration of inflammatory cells and the release of inflammatory mediators in bronchoalveolar lavage fluid (BALF), apart from inhibiting the production of reactive oxygen species (ROS) and lipid peroxidation. Meanwhile, Kin also promoted mitochondrial fusion and restrained mitochondrial fission in mice with ALI. In terms of mechanism, Kin pretreatment increased the phosphorylation of AMP-activated protein kinase (AMPK) and the protein level of nuclear factor erythroid 2-related factor 2 (NRF2). In *Ampk-α* knockout mice challenged with LPS, Kin lost its pulmonary protective effects, accompanied by lower NRF2 level. *In vitro* experiments further unveiled that either AMPK inhibition by Compound C or NRF2 knockdown by siRNA abolished the protective roles of Kin in LPS-treated A549 lung epithelial cells. And NRF2 activator TAT-14 could reverse the effects of *Ampk-α* deficiency. In conclusion, Kin possesses the ability to prevent LPS-induced ALI by modulating mitochondrial dynamic equilibrium in lung epithelial cell in an AMPK/NRF2-dependent manner.

## 1. Introduction

Acute lung injury (ALI) is featured by acute respiratory insufficiency manifested as tachypnea, reduced lung compliance, cyanosis refractory to oxygen, and diffuse alveolar infiltrates [1, 2]. Unless ALI is controlled promptly, it may deteriorate into acute respiratory distress syndrome (ARDS), a life-threatening lung disease with high mortality [3]. ALI could be triggered by a great many factors including infection, ischemia reperfusion, drugs, drowning, trauma, and shock, the pathophysiology of which includes local inflammatory response due to leukocyte infiltration, cytokines and oxidants, epithelial and endothelial injury-mediated increased permeability pulmonary edema, fibrosing alveolitis, and dysregulation of coagulation [4, 5]. The present therapies could not obviously decrease the mortality of patients with ALI or ARDS although great progress has been made in exploring the potential pathophysiology and novel approaches to pharmacotherapy agents [6]. One of the fundamental and critical mechanisms contributing to ALI is oxidative stress, characterized by an impaired induction of antioxidants and imbalance in increased levels of oxidants in lung epithelial cells [7]. During ALI, excessive reactive oxygen species (ROS) in mitochondria can give rise to adenosine triphosphate (ATP) deprivation, loss of mitochondrial DNA integrity, and cytosolic oxidative burst and trigger cell death, indicating that maintaining the homeostasis of mitochondrial dynamics is a prerequisite for lung epithelial function [8–11].

Kinsenoside (Kin) is derived from the traditional plants *Anoectochilus roxburghii*, serving as an active small-molecule natural component employed to combat liver disease, cancers, and hyperglycemia clinically [12–14]. One recent study unveiled that Kin could attenuate tert-butyl hydroperoxide-induced mitochondrial dysfunction in nucleus pulposus cells by activating nuclear factor erythroid 2-related factor 2 (NRF2) [15]. In addition, Kin also alleviated H_2_O_2_-induced oxidative damage by inhibiting ERK/p38/NF-*κ*B signaling in retinal pigment epithelium [16]. However, the possible effects of Kin on LPS-induced ALI remain unclear. With the role of Kin in oxidative stress and epithelium, the hypothesis is raised that Kin may prevent the lung against LPS-induced pathological injury, oxidative stress, and mitochondrial dysfunction. Also, the potential mechanisms by which Kin maintains the homeostasis of mitochondrial dynamics and protects A549 lung epithelial cells were investigated.

## 2. Materials and Methods

### 2.1. Reagents

Kinsenoside (Kin) (# HY-N2292), Compound C (HY-12831), and TAT-14 (HY-P1328) were obtained from MedChemExpress Co., Ltd. (Shanghai, China) with the purity of 99.91%. Lipopolysaccharide (LPS) purified by phenol extraction (0111:B4) was purchased from Sigma-Aldrich LLC (#L2630, Shanghai, China). Primary antibodies against GAPDH (#ab8245), NRF2 (#ab62352), total AMPK (T-AMPK) (#ab207442), and phosphorylated AMPK (P-AMPK) (#ab133448) were purchased from (Cambridge, UK) while secondary antibody was provided by LI-COR Biosciences (Lincoln, United States). Trypsin-EDTA (0.25%) phenol red and Ham's F-12K medium containing 10% fetal bovine serum (FBS) were obtained from Invitrogen-Gibco (Grand Island, NY). All chemical reagents in this study possess analytical grade.

### 2.2. Animals and ALI Model

All animal experiments performed in this study were in accordance with the guidelines of the National Institutes of Health. Male C57BL/6 mice (8-10 weeks, 22-25 g) were provided by the Institute of Laboratory Animal Science, Chinese Academy of Medical Sciences while AMPK*α* knockout mice possessing C57BL/6 background were purchased from Nanjing Gem Pharmatech. The ALI model was established by intratracheal injection of LPS (5 mg/kg) dissolved in 50 *μ*L sterile saline as described previously [17]. Before LPS intratracheal injection, Kin was administered orally (100 mg/kg/day) for 7 consecutive days according to a previous study [18]. Under deep anesthesia, the mice were sacrificed by cervical dislocation after LPS stimulation for 12 h. Subsequently, the intact left lungs were excised and instilled with 10% neutral buffer formalin to keep the alveoli intumescent. Meanwhile, the right lungs were quickly stored into liquid nitrogen for biochemical analysis after they were cut into pieces quickly.

### 2.3. Lung Wet/Dry Ratio

Once the left lung was excised, intrapulmonary blood was washed and removed. And the wet weight of each lung was detected and recorded. Then, these lung tissues were dried using an oven (80°C) for 4 days until the weight kept constant. After that, the dry weight of each lung was recorded and the lung wet/dry ratio was calculated.

### 2.4. H&E Staining

The lung tissues embedded in paraffin were sliced into 4 *μ*m thick sections. Then, haematoxylin and eosin (H&E) was employed to stain lung sections. According to one previous study [19], the lung injury degree was scored to display edema, hemorrhage, and inflammatory cell infiltration. In detail, morphological alterations were scored as severe (3), moderate (2), mild (1), or nil (0) injury according to the presence of exudates, hemorrhage, hyperemia or congestion, infiltration of inflammation, and cellular hyperplasia.

### 2.5. Total Protein and Cytokines in Bronchoalveolar Lavage Fluid (BALF)

The BALF was collected by intratracheal injections of ice-cold PBS (1.0 mL, pH = 7.4) into the lung for three times. The BALF was centrifuged at 400 x g for 15 min at 4°C to obtain cell-free supernatant, which was then stored at −80°C for total protein and proinflammatory cytokines. Then, the total protein and cytokines in BALF were detected using assay kits according to the previous description [20].

### 2.6. Cell Culture and Treatment

The human A549 lung epithelial cell line was selected for in vitro experiment based on a previous study [21], which was purchased from American Type Culture Collection (ATCC, Manassas, VA, USA), which was cultured in Ham's F-12K medium containing 10% FBS at 37°C in a 5% CO_2_ incubator. Compound C was used to inactivate AMPK*α*, and TAT-14 was used to activate NRF2. The expression of NRF2 in A549 lung epithelial cells was knocked down by NRF2 small interfering RNA (siRNA), which was provided by GenePharma Co. Ltd. (Shanghai, China). On the basis of the manufacturer's instructions, the A549 lung epithelial cells were transfected using Lipofectamine™ 3000 Transfection Reagent (Thermo Fisher Scientific, Waltham, MA, USA). The A549 lung epithelial cells were incubated with LPS at the concentration of 100 *μ*g/mL to establish an *in vitro* ALI model with or without Kin (15 *μ*M) for 6 h [22, 23].

### 2.7. Real-Time Quantitative Polymerase Chain Reaction (RT-qPCR)

Total RNA was extracted from murine lung tissues and A549 lung epithelial cells using TRIzol reagent, and transcription-PCR was carried out via real-time PCR. The PCR products were normalized to *Gapdh* mRNA levels and calculated using the 2^-*ΔΔ*Ct^ method. The primer sequences are presented in Table 1.

### 2.8. Western Blot

To begin with, the cells were kept on ice and the tissues from -80°C were lysed by a RIPA buffer (150 mM NaCl, 20 mM Tris-HCl, 1 mM EDTA, 1% sodium deoxycholate, and 1% Nonidet P-40). Then, total proteins were extracted according to the procedure. Subsequently, the protein concentrations were detected by a commercial BCA Protein Assay Kit. And the total proteins were loaded into a 10% sodium dodecyl sulfate-polyacrylamide gel electrophoresis (SDS-PAGE) gel, which were next transferred on a polyvinylidene fluoride (PVDF) membrane. After being blocked with 5% skim milk, the membranes were incubated with P-AMPK (1 : 500 dilution), T-AMPK (1 : 1000 dilution), NRF2 (1 : 1000 dilution), GAPDH (1 : 1000 dilution), and secondary antibodies. At last, the membranes were screened and visualized via an Odyssey Imaging System (Odyssey, LI-COR). The protein levels were normalized to T-AMPK or GAPDH.

### 2.9. Cell Viability

Cell viability was assessed using a commercial CCK-8 assay kit. The A549 lung epithelial cells which were seeded in a 96-well plate were treated with different concentrations of Kin for 6 h. 100 *μ*L of CCK-8 working solution (10%) was then added into the samples for 2 h. At last, the microplate reader was employed to detect the absorbancy of each sample at 450 nm (Bio-Tek®, Synergy HT, Winooski, VT, USA).

### 2.10. Detection of LDH Release in Cell Culture Medium

After LPS stimulation for 6 h, the cell culture medium was collected for LDH detection. To begin with, we prepared the samples for standard curve using LDH assay buffer as well as nicotinamide adenine dinucleotide (NAD) mother liquor. Then, the reaction mix (2 *μ*L of LDH substrate mix and 48 *μ*L of LDH assay buffer) was added into the standard samples or sample to be tested for 1 h in the dark. The microplate reader was employed to detect the absorbancy of each sample at 450 nm (Bio-Tek®, Synergy HT, Winooski, VT, USA).

### 2.11. Determination of Oxidative Stress

The assay kits for the content of malondialdehyde (MDA) (#ab233471, Cambridge, UK) and the activity of nicotinamide adenine dinucleotide phosphate) (NADPH) oxidase (#ab65349, Cambridge, UK) were purchased from Abcam. The assay kit for the activity of superoxide dismutase (SOD) (#A001-3-2, Nanjing, China) was purchased from Nanjing Jiancheng Bioengineering Institute. The activity was determined by selective absorption of light by a solution. The detailed procedures were referred to the manufacturer's instructions.

Also, ROS was detected using fluorescence probe. In detail, fresh lung tissues were washed with PBS for three times. Then, the tissues were embedded in optimum cutting temperature compound and snapfrozen. To block the autofluorescence, AutoFluo quencher was incubated on the frozen sections, followed by the incubation with ROS fluorescence probe away from light for 0.5 h at 37°C. At last, the Olympus DX51 fluorescence microscope was used to observe ROS intensity. Mean ROS intensity is represented by the ratio of integrated density to the area of the region. Data were analyzed by a digital analysis software (Image-Pro Plus 6.0, Media Cybernetics, Bethesda, MD, USA) with 50 cells per slide analyzed for the detection of the area and >10 fields per group assessed for the evaluation of oxidative stress.

### 2.12. Transmission Electron Microscopy

Fresh lung tissues (1 mm × 1 mm × 1 mm) were fixed in certain solution containing 2.5% glutaraldehyde in 0.1 M phosphate buffer (pH 7.4) for 4 hours. Whereafter, the lung tissues were permeated, dehydrated, and embedded in acetone and sectioned approximately at 70 nm. Then, the ultrathin sections were stained with 3% uranyl acetate and lead citrate and viewed under a transmission electron microscope (HITACHI, Japan) at 80 KV. And the number and morphology of mitochondria in lung epithelial cells were observed.

### 2.13. Survival Condition

Another 40 wild-type mice were divided into 4 groups (*n* = 10) to record their survival condition. The death number of mice was recorded at 17:00 every day. The survival rate within 7 days was calculated after LPS and/or Kin intervention. Survival conditions among the 4 groups were compared via Kaplan-Meier survival curves using a log-rank test.

### 2.14. Statistical Analysis

All data in this study are presented as mean ± standard deviation (means ± SD) and analyzed via the SPSS 22.0 statistical software. The differences between the 2 groups were compared using an unpaired, two-sided Student's *t*-test while the differences among 3 or more groups were compared using one-way ANOVA followed by a post hoc Tukey's test. Kaplan-Meier method was carried out to investigate the survival rates, and the survival distributions between four groups were compared using a log-rank test. *P* < 0.05 was regarded as statistical significance.

## 3. Results

### 3.1. Kin Alleviated LPS-Induced Lung Injury and Improved Survival of Mice

**Figure 1:**
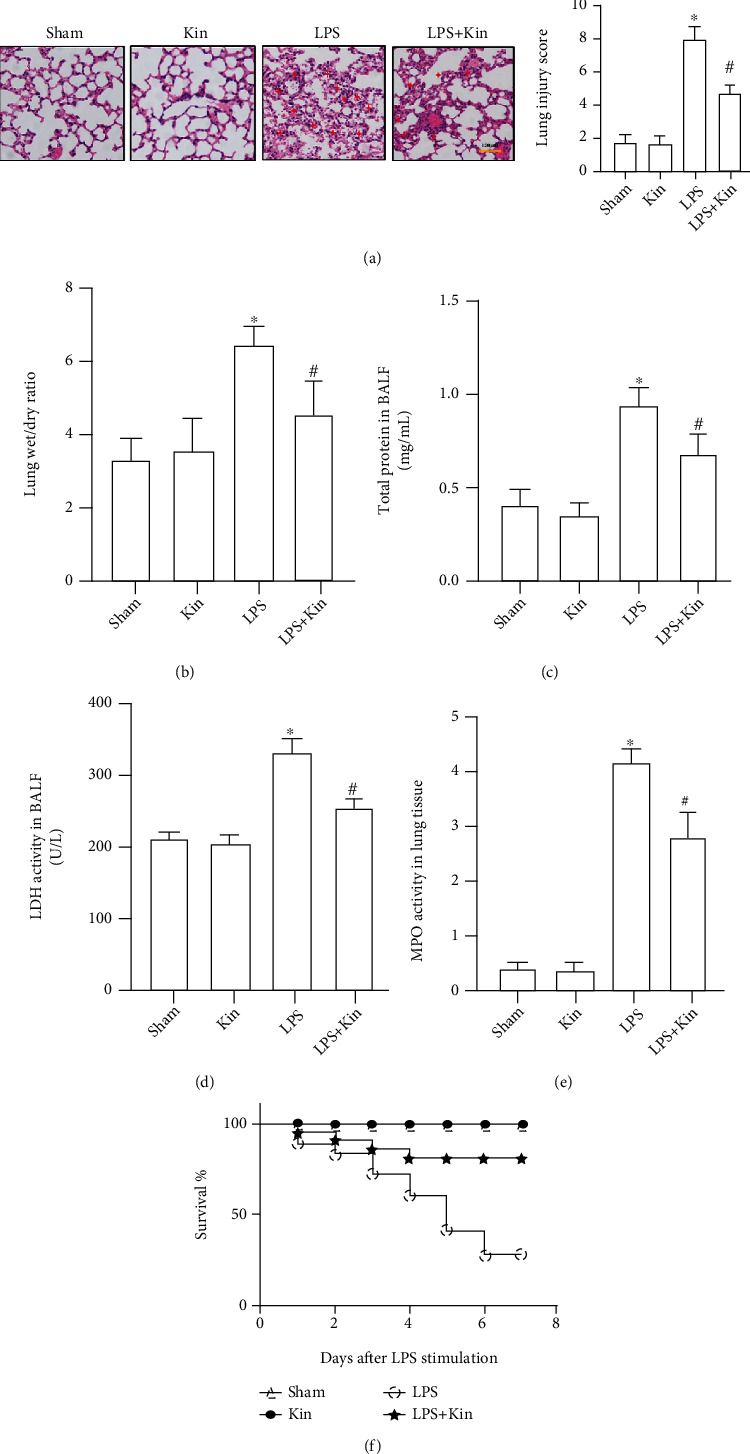
Attenuation of LPS-induced ALI in mice treated with Kin (100 mg/kg). (a) Representative images of H&E staining of lung tissues and lung injury score (*n* = 5), scale bars, 100 *μ*m. (b) The lung wet-to-dry ratio (*n* = 5). (c) Total protein in BALF from mice in the indicated groups (*n* = 5). (d) LDH activity in BALF from mice in the indicated groups (*n* = 5). The data are expressed as the mean ± SD. ^∗^*P* < 0.05 (versus Sham group); #*P* < 0.05 (versus LPS group). (e) MPO activity in lung tissues (*n* = 5). (f) 7-day survival curve of LPS (10 mg/kg)-treated mice (*n* = 10). ^∗^*P* < 0.05 (versus Sham group); #*P* < 0.05 (versus LPS group).

H&E staining showed that LPS stimulation significantly aggravated pathological injury, as evidenced by pulmonary edema, hemorrhage, and inflammatory cell infiltration, which could be partially reversed after Kin pretreatment (Figure 1(a)). Also, Kin pretreatment also decreased the lung wet-to-dry ratio in mice challenged with LPS (Figure 1(b)). Meanwhile, total protein and LDH activity in BALF in mice challenged with LPS could also be lower after pretreatment with Kin (Figures 1(c) and 1(d)). Additionally, the higher level of MPO activity in lung tissues from mice challenged with LPS was significantly inhibited by Kin (Figure 1(e)). Survival analysis showed that Kin pretreatment significantly improved the 7-day survival rate of mice challenged with LPS (Figure 1(f)). Collectively, Kin displayed obvious protective effects against LPS-induced injury.

### 3.2. Kin Inhibited Inflammatory Response Triggered by LPS in Lung Tissues

**Figure 2:**
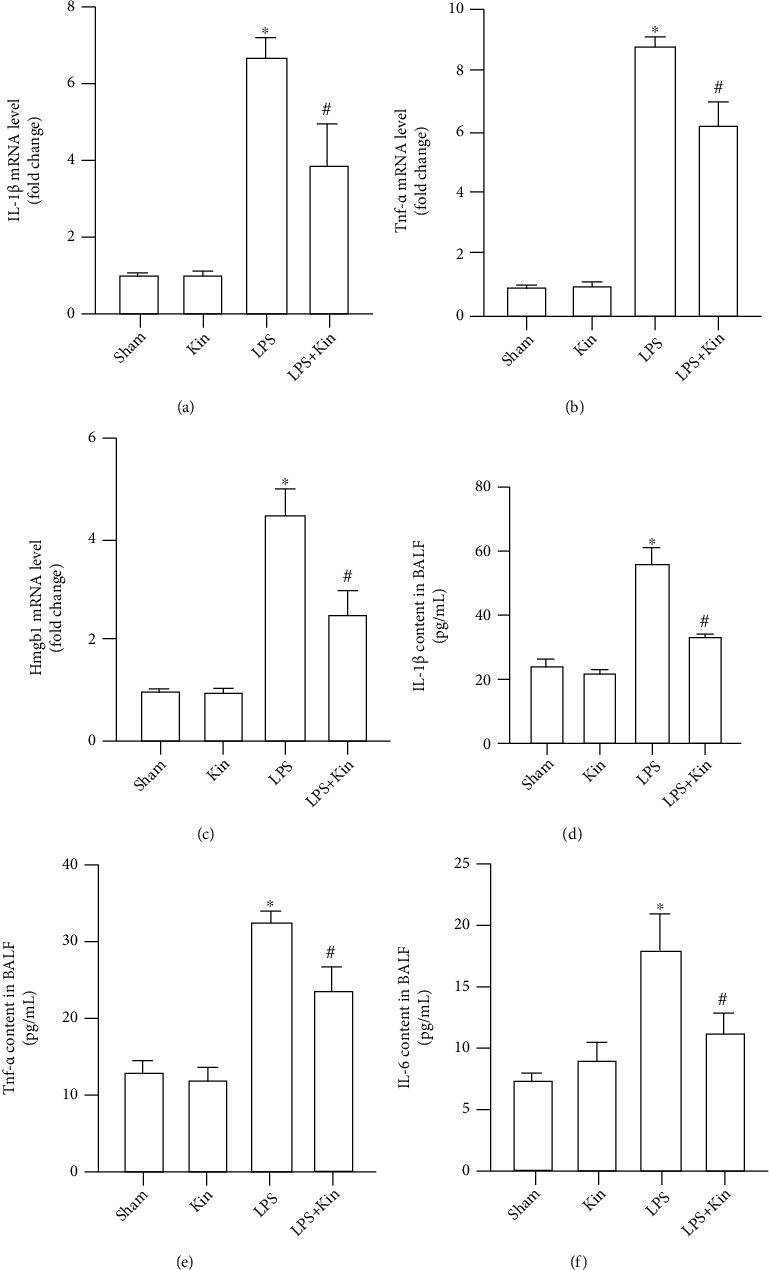
Inhibition of inflammatory response in lung tissues and BALF by Kin (100 mg/kg). (a–c) The mRNA levels of IL-1*β*, Tnf-*α*, and Hmgb1 in lung tissues (*n* = 5). (d–f) The content of IL-1*β*, TNF-*α*, and IL-6 in BALF (*n* = 5). The data are expressed as the mean ± SD. ^∗^*P* < 0.05 (versus Sham group); #*P* < 0.05 (versus LPS group).

Acute inflammation is one of the critical features of LPS-induced lung injury. During acute inflammation, neutrophil and macrophage infiltration into inflammatory sites could produce vast proinflammatory cytokines, mediating subsequent local damage and systemic inflammation [24]. We next detected the levels of proinflammatory cytokines including IL-1*β*, TNF-*α*, IL-6, and HMGB1 in lung tissues and BALF. As shown in Figures 2(a)–2(c), compared with the LPS group, the mRNA levels of IL-1*β*, TNF-*α*, and HMGB1 in the LPS+Kin group were significantly lower. In keeping with this, the protein levels of IL-1*β*, TNF-*α*, and IL-6 in BALF were also lower in the LPS+Kin group than those in the LPS group (Figures 2(d)–2(f)).

### 3.3. Kin Relieved Oxidative Damage Caused by LPS in Lung Tissues

**Figure 3:**
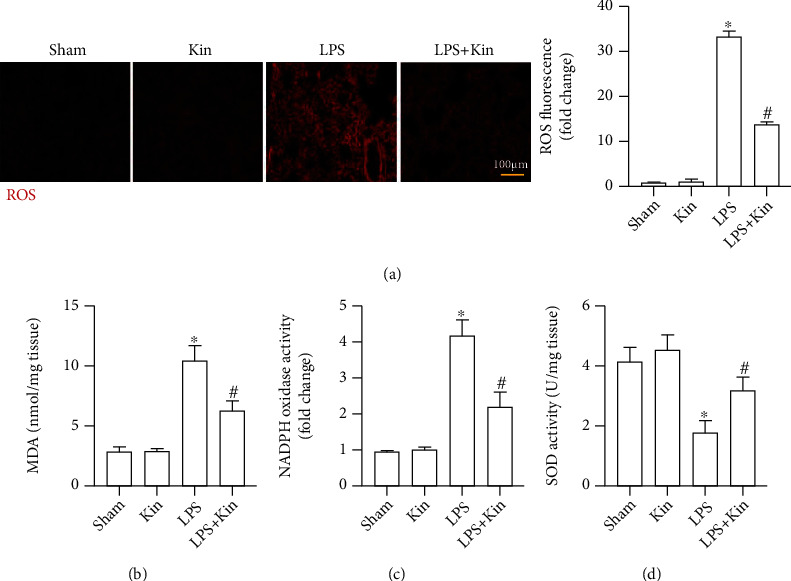
Remission of oxidative stress in lung tissues by Kin (100 mg/kg). (a) Representative images of ROS probe staining of lung tissues and semiquantitative analysis (*n* = 5, 10 + fields per lung), scale bars, 100 *μ*m. (b) The MDA content in lung tissues (*n* = 5). (c) NADPH oxidase activity in lung tissues (*n* = 5). (d) SOD activity in lung tissues (*n* = 5). The data are expressed as the mean ± SD. ^∗^*P* < 0.05 (versus Sham group); #*P* < 0.05 (versus LPS group).

Next, we assessed the level of oxidative stress in the four groups. As shown in Figure 3(a), Kin could significantly inhibit LPS-induced ROS production in lung tissues. Meanwhile, Kin also decreased MDA content and NADPH oxidase activity but enhanced SOD activity in lung tissues from mice challenged with LPS (Figures 3(b)–3(d)). These results indicated that LPS-induced oxidative damage could be mitigated after Kin pretreatment.

### 3.4. Kin Preserved the Dynamic Equilibrium of Mitochondrial Fusion/Fission in Lung Tissues from LPS-Treated Mice

**Figure 4:**
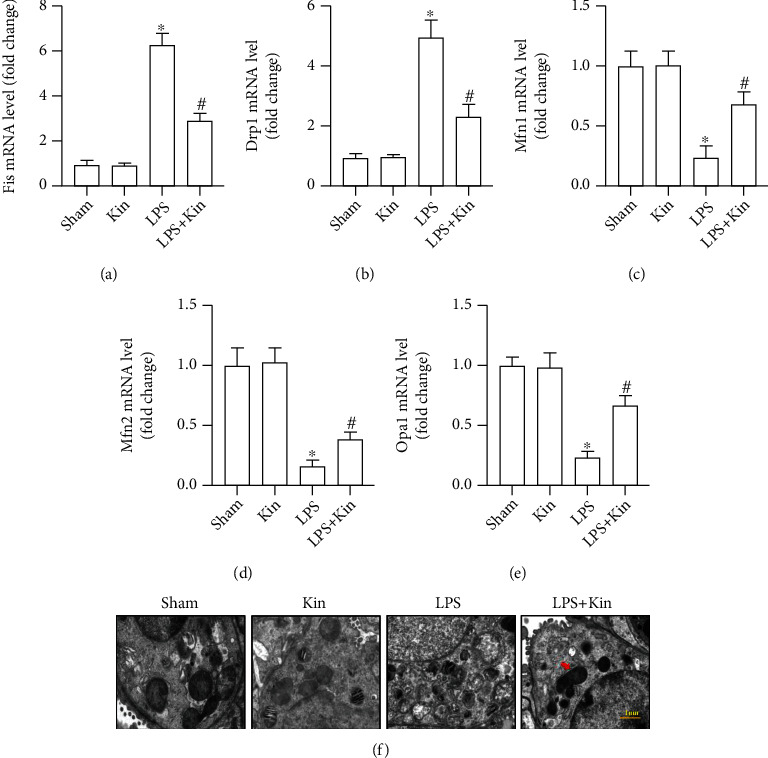
Kin (100 mg/kg) preserved the dynamic equilibrium of mitochondrial fusion/fission in lung tissues from LPS-treated mice. (a–e) The mRNA levels of Fis, Drp1, Mfn1, Mfn2, and Opa1 in lung tissues (*n* = 5). (f) The morphology and number of mitochondria in murine lung epithelial cells under the transmission electron microscopy. The data are expressed as the mean ± SD. ^∗^*P* < 0.05 (versus Sham group); #*P* < 0.05 (versus LPS group).

To further explore the protective effects of Kin on mitochondria, we assessed the condition of mitochondrial fusion/fission in 4 groups by detecting the mRNA levels of Fis, Drp1, Mfn1, Mfn2, and Opa1. As indicated, LPS stimulation significantly inhibited mitochondrial fusion as evidenced by the higher mRNA levels of Fis and Drp1 (Figures 4(a) and 4(b)). Meanwhile, LPS stimulation also significantly decreased the mRNA levels of Mfn1, Mfn2, and Opa1, suggestive of mitochondrial fission (Figures 4(c)–4(e)). As shown in Figure 4(f), LPS stimulation significantly increased the number of damaged mitochondria in murine lung epithelial cells compared with the mice from the Sham group. However, Kin pretreatment could decrease the number of damaged mitochondria and promote mitochondrial fusion. Collectively, Kin pretreatment obviously shifted the balance towards a fusion phenotype.

### 3.5. AMPK/NRF2 Pathway May Be Involved in the Protective Effects of Kin on Mitochondrial Dynamics after LPS Challenge

**Figure 5:**
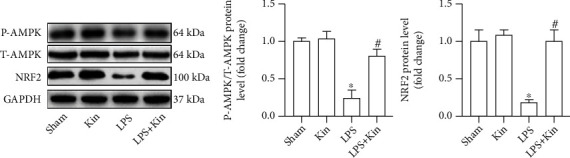
AMPK/NRF2 pathway may be involved in the protective effects of Kin (100 mg/kg) on mitochondrial dynamics after LPS challenge. Representative Western blots and quantitative results of phosphorylated AMPK (P-AMPK), total AMPK (T-AMPK), and NRF2 in lung tissues (*n* = 5). The data are expressed as the mean ± SD. ^∗^*P* < 0.05 (versus Sham group); #*P* < 0.05 (versus LPS group).

AMPK is one of the core molecules regulating energy metabolism, which is essential for cell homeostasis. The activation of AMPK could preserve the dynamic equilibrium of mitochondrial fusion/fission by regulating NRF2 [25]. Western blot showed that LPS stimulation significantly inhibited the phosphorylation of AMPK and decreased the protein level of NRF2 in murine lung tissues. After Kin pretreatment, the phosphorylation of AMPK and the protein level of NRF2 in LPS-treated lung tissues were higher (Figure 5), hinting that Kin may exert pulmonary protection by activating the AMPK/NRF2 pathway.

### 3.6. *Ampk-α* Deficiency Abolished the Protective Effects of Kin in Mice Challenged with LPS

**Figure 6:**
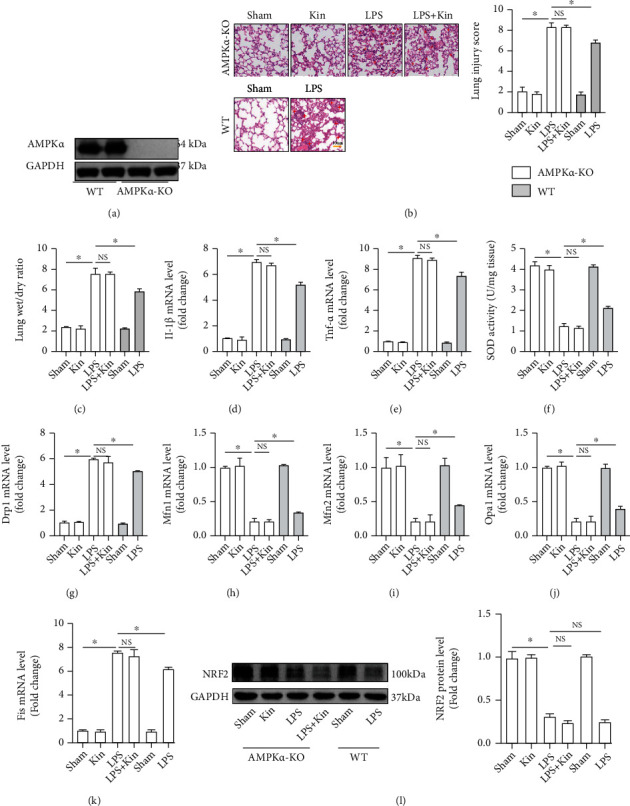
AMPK*α* deficiency abolished the protective effects of Kin (100 mg/kg) in mice challenged with LPS. (a) Representative Western blots of AMPK*α* in wild-type mice and AMPK*α* knockout mice. (b) Representative images of H&E staining of lung tissues from AMPK*α* knockout mice and wild-type mice (*n* = 5), scale bars, 100 *μ*m. (c) The lung wet-to-dry ratio (*n* = 5). (d, e) The mRNA levels of IL-1*β* and Tnf-*α* in lung tissues (*n* = 5). (f) SOD activity in lung tissues (*n* = 5). (g–k) The mRNA levels of Drp1, Mfn1, Mfn2, Opa1, and Fis in lung tissues (*n* = 5). (l) Representative Western blots and quantitative results of NRF2 (*n* = 5). The data are expressed as the mean ± SD. ^∗^*P* < 0.05 (versus the indicated groups); NS: no statistical significance.

To further determine that the protective effects of Kin in ALI were mediated in an AMPK/NRF2 pathway-dependent manner, we next used *Ampk-α* global knockout mice to prove our hypothesis.  Figure 6(a) displays the protein expression of AMPK-*α* in lung tissues from wild-type mice and *Ampk-α* global knockout mice. H&E staining (Figure 6(b)) demonstrated that in AMPK*α*-deficient mice, Kin pretreatment could not alleviate LPS-induced lung injury. The lung wet/dry ratio of *Ampk-α* global knockout mice showed no significant difference between the LPS group and the LPS+Kin group (Figure 6(c)). RT-PCR also unveiled that the Kin could not reverse the increase of IL-1*β* and Tnf-*α* induced by LPS after AMPK-*α* was inhibited (Figures 6(d) and 6(e)). Additionally, *Ampk-α* deficiency also prevented the antioxidative effects of Kin in murine lung tissues challenged with LPS (Figure 6(f)). Next, we detected the mitochondrial fusion/fission in the indicated groups. As shown in Figures 6(g)–6(k), Kin lost the ability to preserve the dynamic equilibrium of mitochondrial fusion/fission in *Ampk-α*-deficient mice challenged by LPS. Last but not the least, Kin could not activate NRF2 in LPS-treated mice after *Ampk-α* was inhibited (Figure 6(l)).

### 3.7. Kin Could Protect against LPS-Induced A549 Lung Epithelial Cell Injury by Modulating Mitochondrial Biogenesis in an AMPK/NRF2 Pathway-Dependent Manner

**Figure 7:**
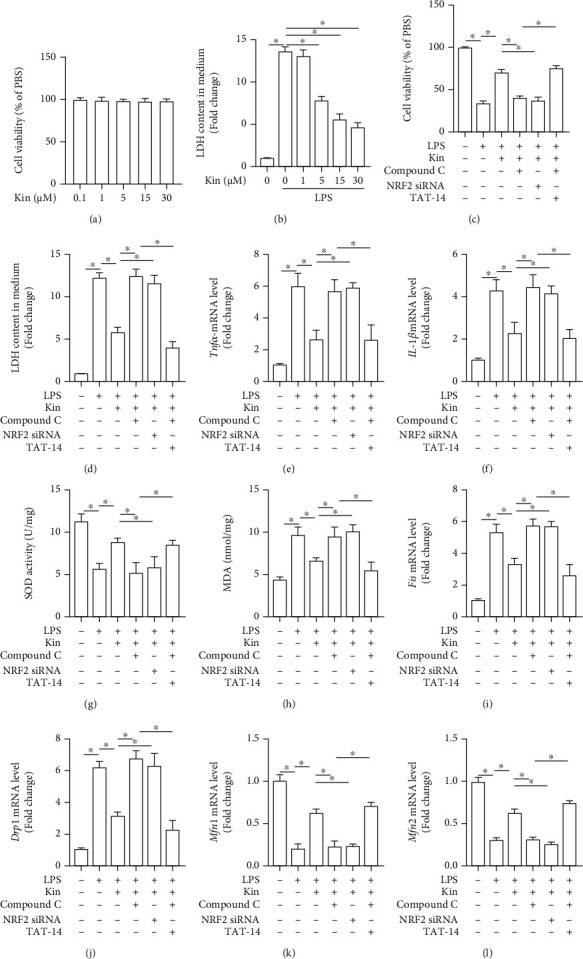
Kin could protect against LPS-induced lung epithelial cells injury by modulating mitochondrial biogenesis in an AMPK/NRF2 pathway-dependent manner. (a) Cell viability of lung epithelial cells treated with different concentrations of Kin (*n* = 6). ^∗^*P* < 0.05 (versus 0.1 *μ*M groups). (b) LDH content in medium from LPS-induced lung epithelial cells treated with different concentrations of Kin (*n* = 6). ^∗^*P* < 0.05 (versus 0.1 *μ*M groups). (c) Cell viability of lung epithelial cells in the indicated groups (*n* = 6). (d) LDH content in medium in the indicated groups (*n* = 6). (e, f) The mRNA levels of Tnf-*α* and IL-1*β* in the indicated groups (*n* = 6). (g, h) The markers of oxidative stress in the indicated groups (*n* = 6). (i–l) The markers of mitochondrial fusion and fission in the indicated groups (*n* = 6). All data are expressed as the mean ± SD. The significance between these groups was tested, control group and LPS group, LPS group and LPS+Kin group, LPS+Kin group and LPS+Kin+Compound C group, LPS+Kin group and LPS+Kin+NRF2 siRNA group, and LPS+Kin+Compound C group and LPS+Kin+Compound C group+TAT-14. ^∗^*P* < 0.05 compared with the indicated groups.

To verify the mechanism by which Kin prevented LPS-induced ALI, we treated A549 lung epithelial cells with the AMPK inhibitor Compound C, the NRF2 siRNA, and the NRF2 activator TAT-14. To begin with, we detected cell viability of A549 lung epithelial cells treated with different concentrations of Kin (0.1-30 *μ*M). As shown in Figure 7(a), none of five concentrations of Kin could affect the viability of A549 lung epithelial cells. However, the concentration of 5, 15, and 30 *μ*M of Kin could decrease LDH release in LPS-treated A549 lung epithelial cells (Figure 7(b)). Hence, we next selected the concentration of 15 *μ*M for the subsequent *in vitro* experiments. As expected, AMPK inhibition or NRF2 knockout significantly abolished cytoprotection from Kin in LPS-treated A549 lung epithelial cells, as indicated by the decreased cell viability and the increased LDH content. However, NRF2 activation by TAT-14 reversed the effects of Compound C (Figures 7(c) and 7(d)). Similarly, Kin also inhibited inflammatory response and oxidative stress induced by LPS *in vitro* in an AMPK/NRF2 pathway-dependent manner (Figures 7(e)–7(h)). Kin shifted mitochondrial dynamics from fission to fusion in LPS-treated A549 lung epithelial cells, which could be counteracted after AMPK or NRF2 inhibition. And TAT-14 could reverse the effects of AMPK inhibition on LPS-treated A549 lung epithelial cells treated with Kin (Figures 7(i)–7(l)). Taken together, these data further demonstrated that the protective effects from Kin on LPS-treated A549 lung epithelial cells were mediated by the AMPK/NRF2 pathway.

## 4. Discussion

**Figure 8:**
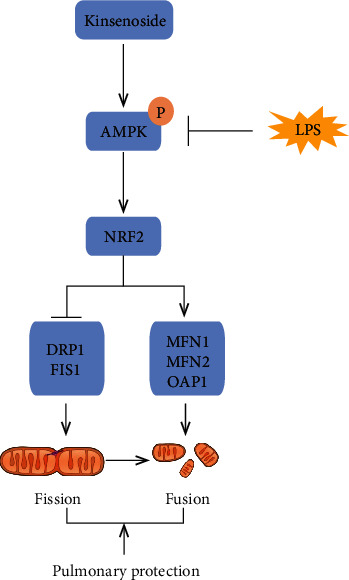
A putative scheme illustrating the mechanism by which Kin prevents LPS-induced ALI via activation of the AMPK/NRF2 signaling pathway.

In this study, we disclosed that Kin pretreatment could alleviate LPS-induced ALI in mice. The possible mechanisms may be associated with Kin's role in preserving the dynamic equilibrium of mitochondrial fusion/fission in A549 lung epithelial cells (Figure 8). Hence, Kin pretreatment owns the potential to combat sepsis-induced ALI in the future.

Kin, serving as a compound extracted from medicinal herb *Anoectochilus formosanus*, possesses versatile pharmacological actions including anti-inflammation, antioxidative stress, and immunoregulation in various acute diseases and chronic diseases involving diabetes, hypertension, nephritis, osteoarthritis, and autoimmune hepatitis [12, 18, 26]. However, the relevance of Kin in ALI and sepsis has not been reported yet. In the current study, Kin pretreatment dramatically alleviated pathological injury, edema, and inflammatory response as oxidative stress in lung tissues from LPS-treated mice and LPS-induced A549 lung epithelial cells.

Mitochondria is the most critical organelle responsible for the generation of ATP. When cells are subjected to environmental or metabolic stress, mitochondria will start delicate fission and fusion cycles to preserve their functions to the full extent [27, 28]. Mitochondrial quality control could improve the ability of lung cells to replace and eliminate damaged mitochondria, supporting cell survival [29]. On the contrary, substantial mitochondrial damage could contribute to epithelial barrier dysfunction by multiple mechanisms including calcium dysregulation, energy failure, disturbance of heme homeostasis, and regulated cell death [30]. It is worth noting that notably, the dynamics of mitochondria are usually initiated by certain prime guanosine-5′-triphosphatases from the dynamin family. In mammals, the fission process is initiated and regulated by Fis and Drp1 while the fusion process is governed by Mfn1, Mfn2, and Opa1. The homeostasis and dynamic equilibrium of mitochondria are the preconditions for its functional integrity [31]. Once the dynamic equilibrium is impaired, cellular metabolic and energy disorder will be triggered, contributing to cell death and various tissue damage [32]. During sepsis, mitochondrial dysfunction is one of the critical mechanisms resulting in organ injury and failure. Previous studies have unveiled that several signaling pathways were associated with the dynamic equilibrium of mitochondria during ALI. For instance, the activation of heme oxygenase-1 (HO-1)/carbon monoxide by the PI3K/Akt pathway could maintain mitochondrial dynamic equilibrium in the context of lipopolysaccharide [9, 10]. Additionally, adiponectin deficiency could also induce mitochondrial dysfunction by inhibiting SIRT3 and PGC1*α* at baseline, thus increasing the susceptibility to LPS-induced ALI [33]. In distinct alveolar cells, Suliman et al. clarified the association between mitochondrial biogenesis and the death of lung epithelial cells. And they found that substantial mitochondrial damage happened preceding the death of lung epithelial cells [34]. Here, our results unveiled that Kin pretreatment significantly decreased the mRNA levels of Fis and Drp1 but increased the levels of Mfn1, Mfn2, and Opa1 in LPS-treated mice and A549 lung epithelial cells, hinting that Kin owned the ability to shift the balance from a fission towards a fusion phenotype.

AMPK is one of the crucial energy sensors which could modulate mitochondrial biogenesis through activating NRF2 in this phosphorylated status [35]. Once NRF2 is activated, it could translocate into cell nucleus and transcriptionally activate a great many antioxidant genes that are responsible for mitochondrial fusion and fission [25]. Hence, activating the AMPK/NRF2 pathway is a promising strategy to preserve mitochondrial function. In this study, *Ampk-α* deficiency or NRF2 inhibition could abolish the protective roles of Kin in ALI, indicating that Kin may function in an AMPK/NRF2 pathway-dependent manner. However, how Kin activates and promotes the phosphorylation of AMPK needs exploring in the future.

In conclusion, we disclosed that Kin alleviated LPS-induced ALI by shifting mitochondria from fission to fusion through the AMPK/NRF2 pathway in A549 lung epithelial cells. The present study may have potential for the future prevention of ALI and sepsis through the application of Kin.

## Figures and Tables

**Table 1:** The primer sequences in the study.

Species	Gene	Forward primer	Reverse primer
Mice	*Tnf-α*	ACTGAACTTCGGGGTGATCGGT	TGGTTTGCTACGACGTGGGCTA
Mice	*Hmgb1*	CGATGCTGATGCTGAATAGCC	CCAGCTGATGTAGTCGTAGTCA
Mice	*Il-1β*	AATGAAGGAACGGAGGAGCC	CTCCAGCCAAGCTTCCTTGT
Mice	*Gapdh*	ACTCCACTCACGGCAAATTC	TCTCCTATGGTGGTGACGACA
Mice	*Fis*	ATTCGTAGCTAGTCGTAAACC	TCGTAGTCGTAGTAGTGCTACC
Mice	*Drp1*	CCCGTAGTCGTAGATGCTGAT	CGATGCTGATCGTATGTCGTAA
Mice	*Mfn1*	TTGCTAGTCGTAGCTGATGCC	CCGTAGCTAGTCGTAGTCGTACC
Mice	*Mfn2*	AGCTAGTGCTTCGTAGTCGTAG	ACCCGTAGTCGATGCAGATCGTA
Mice	*Opa1*	TTACGTAAACGTAGCTGTTACC	CAGCTGATGCTGATGCTGAATTT
Human	*Tnf-α*	CGTAGCTGATGAAGCTGATGC	TGTCGATGCTGAAACAGTGCTAGT
Human	*Il-1β*	AACTGCTGATGTCGTAGTCGA	ACGATGTCGATGTCGTGTAGC
Human	*Fis*	CGATGCTGATGCTGAAACGTAGC	CATCGTAGTCAAACGTACTTACCC
Human	*Drp1*	CGATGCTGATGCTGATTATAGTCA	CTTAGTCGTGTACGTAGTCGCCAA
Human	*Mfn1*	AACGTAGTCGATACCAACGTA	CCTAGTCGTAAGATGCTGATCGTA
Human	*Mfn2*	ACTGTAGTCCAGCTGATGCTAA	AAATTGTCGATGCCCTGATCGATC
Human	*Gapdh*	TGCTGATGCTGATGTCGATCCC	CACATGCTGATGATACAACTG

## Data Availability

All data supporting the findings in our study are available from the corresponding author upon reasonable request.
